# Comparative post-hatching histogenesis of gallbladder and extrahepatic bile ducts in chickens, ducks, and pigeons

**DOI:** 10.3389/fvets.2026.1969438

**Published:** 2026-10-30

**Authors:** Nazema S. Abdel-Megeid, Liana Fericean, Sami F. Mahmoud, Ahmed Elweza, Bănăţean-Dunea Ioan, Mihaela Ostan, Olga Rada, Ahmed Magdy, Mohamed Abdo

**Affiliations:** 1 Department of Histology and Cytology, Faculty of Veterinary Medicine, Menoufia University, Shibin El-Kom, Egypt; 2 Veterinary Medicine Program, Menoufia National University, Shibin El-Kom, Egypt; 3 Department of Biology and Plant Protection, Faculty of Agriculture, University of Life Sciences “King Michael I” from Timișoara, Timisoara, Romania; 4 Research Center of Basic Sciences, Engineering and High Altitude, Taif University, Taif City, Saudi Arabia; 5 Department of Biotechnology, College of Science, Taif University, Taif City, Saudi Arabia; 6 Department of Theriogenology, Faculty of Veterinary Medicine, University of Sadat City, Sadat City, Menofia, Egypt; 7 Department of Animal Histology and Anatomy, School of Veterinary Medicine, Badr University in Cairo (BUC), Badr City, Egypt; 8 Department of Anatomy and Embryology, Faculty of Veterinary Medicine, University of Sadat City, Sadat City, Egypt

**Keywords:** chicken, duck, gallbladder, histogenesis, pigeon, post-hatching development

## Abstract

**Introduction:**

The post-hatching development of the gallbladder and extrahepatic bile ducts play an important role in the functional maturation of the avian digestive system. However, post-hatching histogenesis is poorly characterized among species. This comparative study investigated the histogenesis of these biliary structures in chickens (*Gallus gallus* domesticus), ducks (*Anas platyrhynchos*) and pigeons (*Columba livia*) from one day after hatching to eight months of age.

**Methods:**

Tissue samples were examined from Forty birds (*n* = 16,16,8 for each age group) of domestic chickens, ducks and pigeons at four post-hatching stages; Stage I: first day (1 dph); Stage II: one month post hatching (1 mph), Stage III: 4 months (4 mph) and Stage IV: 8 months (8 mph). The gallbladder and extrahepatic bile ducts were examined by gross morphology and light microscopy with H&E, Masson’s trichrome, Wiegert’s elastic tissue Stain, and combined PAS/Alcian blue (pH 2.5) staining.

**Results:**

The gallbladder was present in chicken and duck but absent in pigeon. Morphologically it was elongated and tubular in chickens, while ovoid in ducks occupying a deep cystic fossa. Three extra-hepatic bile ducts were visualized: Hepato-cystic, Cystico-enteric and Hepato-enteric. Histologically, in ducks, the mucosal villi were consistently shorter and broader, whereas chickens exhibited taller and more elongated villi. During post-hatching development, both species showed progressive maturation of the gallbladder mucosa, characterized by increased villous complexity, progressive stromal collagenization, and lymphocytic infiltration. The lining epithelium showed strong PAS and Alcian blue positivity at all examined post-hatching stages in both species, indicating persistent mucin production and a constitutive mucosal protective barrier.

**Discussion:**

This study provides baseline information on the normal post-hatching development of the avian biliary system and share in comparative anatomy, developmental biology and veterinary pathology as well as serving as a fruitful reference for future studies on avian hepatobiliary health and disease.

## 1 Introduction

Gall Bladder: The gall bladder is a musculo-membranous organ that stores and concentrates bile. This structure is present in most avian species but is notably absent in pigeons, doves, sparrows and ostriches (1, 2). This anatomical variation is often linked to feeding frequency and dietary habits (3). Gallbladder morphology has considerable interspecies variation, in the domestic fowl it is usually described as pear-shaped or cylindrical, while in aquatic birds, e.g., the duck, it is ovoid or spheroid and occupies a deep cystic fossa. In the other hand, it is tubular and elongated in geese and turkeys and rounded in owls (4, 5). Its morphology has been further described in guinea fowl and gulls by recent studies (6, 7). Birds with a gallbladder have usually two extrahepatic bile ducts, a Cystico-enteric duct from the gallbladder and a hepato-enteric duct from the liver, both opening into the duodenum. Double hepato-enteric ducts compensate for the absence of gallbladders in species (8, 9). Histologically, the avian gallbladder wall consists of mucosa, submucosa, muscularis, and serosa. The mucosa is lined with a simple columnar epithelium with villus-like folds and PAS-positive mucin, suggesting a protective role (10). The lamina propria consists of diffuse lymphatic tissue and the muscularis is predominantly circular. Adult histology is well described in chickens, geese, guinea fowl and quail, but data on the post hatching development are scarce (11, 12). Current knowledge of histogenesis of the gallbladder is largely based on embryonic studies. The epithelium is well developed in 15-day-old chick embryo, and neutral mucopolysaccharides are found on day 14, whereas sulfated mucins are found on day 15. However, little is known about the maturation of the gallbladder mucosa, stroma and muscularis during the post-hatching period (13, 14). Recent comparative studies have shown species-specific adaptations in biliary physiology, but a systematic timeline of post-hatching histological changes is not available. The first 8 months post-hatching are considered a critical period of digestive system maturation in poultry, thus knowledge of gallbladder development during this period is necessary to interpret biliary pathology and to develop avian models of gallbladder disease (15, 16). Therefore, this recent study aimed to: establish the first comparative histological timeline of gallbladder development in domestic chickens and ducks from 1 day to 8 months post-hatching and compare these data with pigeons that lack a gallbladder to explore the structural basis of their alternative biliary drainage. To our knowledge, no previous study has systematically tracked post-hatching changes in mucosal complexity, stromal composition, and mucin histochemistry across these species.

## 2 Materials and methods

### 2.1 Ethics statement

All experimental procedures were approved by and followed the guidelines of the Scientific Research Ethics and Animal Care Committee (SREACC), Faculty of Veterinary Medicine, Menoufia University. The ethical approval code was MU/Vet. Med-2026/002.

### 2.2 Animals and experimental design

A total of 40 clinically healthy birds of both sexes were used: domestic chickens (*Gallus gallus* domesticus, *n* = 16), ducks (*Anas platyrhynchos*, *n* = 16), and pigeons (*Columba livia*, *n* = 8). Birds were obtained from commercial poultry farms in Menoufia Governorate, Egypt, and housed under standardized conditions (23 ± 2 °C, 12 h light/dark cycle) with ad libitum access to water and a commercial diet. Birds were assigned to four post-hatching age groups (*n* = 4 chickens, *n* = 4 ducks, *n* = 2 pigeons per group):1-day post-hatching (1 dph)1-month post-hatching (1 mph),4 months post-hatching (4 mph),8 months post-hatching (8 mph). Birds were euthanized by cervical dislocation following light ether anesthesia, in accordance with AVMA Guidelines for the Euthanasia of Animals (2020). Post-mortem examination confirmed absence of gross lesions or biliary disease.

### 2.3 Gross morphological examination

The coelomic cavity was opened immediately post-mortem. The topography, shape, and color of the gallbladder and extrahepatic bile ducts were recorded *in situ*. The pathways of the hepato-cystic, Cystico-enteric, and hepato-enteric ducts, and their relationship to the pancreatic ducts and duodenal papillae, were documented. Specimens were photographed using a digital camera (Canon EOS 250D) with a millimeter scale bar for calibration.

### 2.4 Histological and histochemical study

Gallbladder samples were collected within 5 min post-mortem and fixed in 10% neutral buffered formalin for 48 h at room temperature. After fixation, tissues were dehydrated in graded ethanol (70–100%), cleared in methyl benzoate, and embedded in paraffin wax (Sigma-Aldrich). Serial sections of 5 μm thickness were cut using a rotary microtome (Leica RM2125 RTS). Sections of gallbladder in all age groups were stained as follows: Harris’s Hematoxylin and Eosin (H&E) for general histology; Masson’s Trichrome for collagen and smooth muscle fibers; Weigert’s elastic stain for elastic fibers; Gomori’s Reticulin for reticular fibers; Periodic Acid-Schiff (PAS) for neutral mucopolysaccharides; Alcian Blue (AB) pH 2.5 for acidic mucopolysaccharides; Combined AB pH 2.5/PAS for simultaneous detection of acidic and neutral mucins. All staining protocols followed Bancroft and Gamble (17). Digital images were captured using a Leica DM500 microscope equipped with a Leica ICC50 HD camera and 40x/0.65 NA, 100x/1.25 NA oil objectives. Images were acquired using Leica Application Suite v4.12 software at a resolution of 2,592 × 1944 pixels. All measurements were calibrated using a stage micrometer.

### 2.5 Morphometry and statistical analysis

Epithelial height, villous length, and muscularis thickness were measured at 10 random fields per section using ImageJ software (NIH, v1.53). Data is presented as mean ± SD. Differences among age groups were analyzed by one-way ANOVA followed by Tukey’s post-hoc test using SPSS v26. *p* < 0.05 was considered significant.

## 3 Results

### 3.1 Gross morphology of the gallbladder and extrahepatic bile ducts

#### 3.1.1 Chicken (*Gallus gallus* domesticus)

In all examined chickens of all age groups (*n* = 16) the gallbladder was constantly observed. It was situated in the cranial half of the visceral surface of the right liver lobe. Morphologically it was elongated and tubular, its apex directed caudally, presenting a dark green aspect due to bile content (Figures 1a,d,g, 2A; Table 1). The extrahepatic biliary system was composed of a hepato-cystic duct, which connected the right hepatic duct to the gallbladder, and a cystico-enteric duct arising from the neck of the gallbladder and entering the duodenum. A separate hepato-enteric duct arose from the right liver lobe and ran parallel to the cystico-enteric duct. Both cystico-enteric and hepato-enteric ducts, along with two pancreatic ducts, opened on a common papilla in the distal part of the ascending duodenum (Figure 2d).

**Figure 1:**
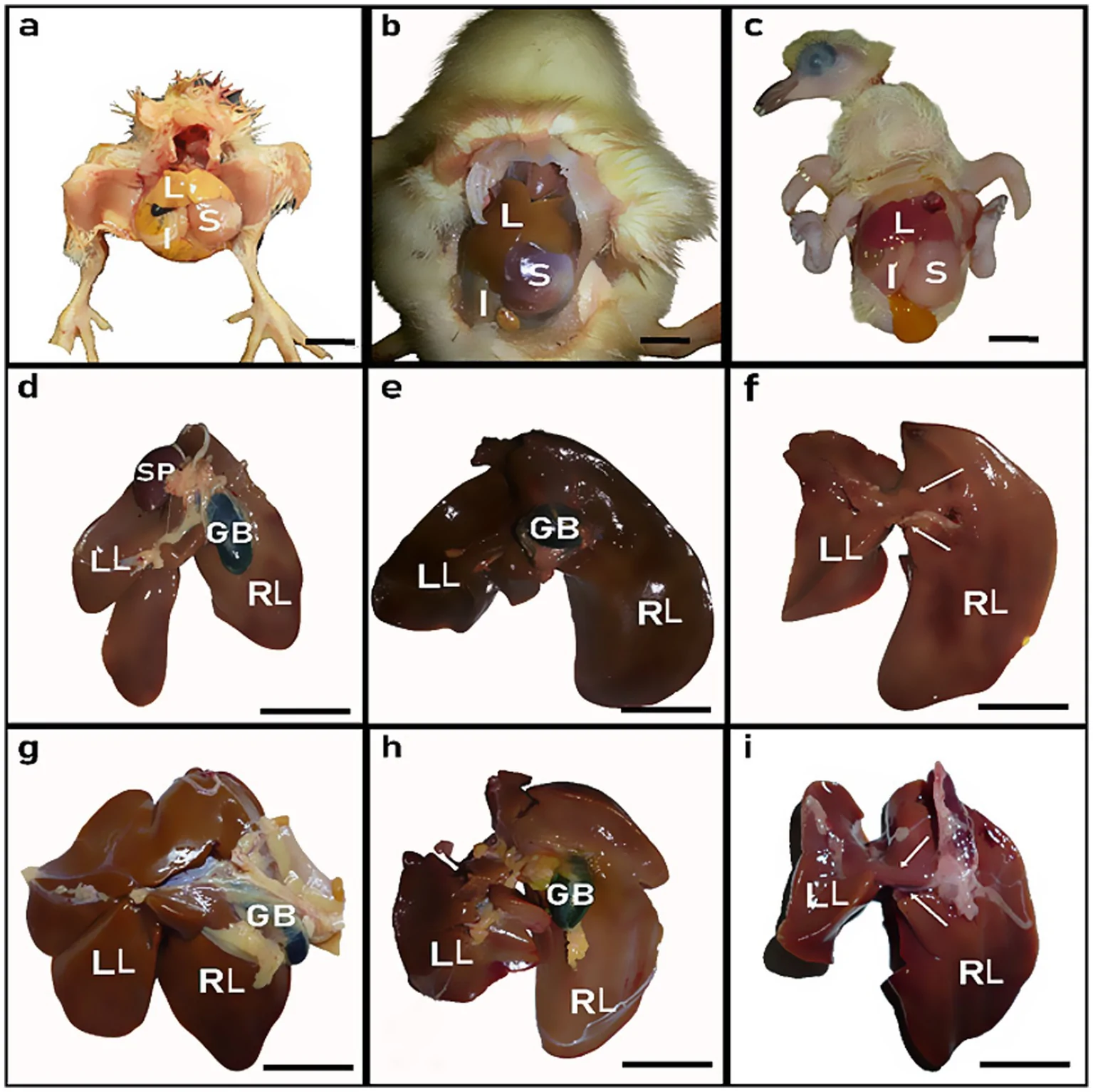
Photograph of the liver showing the gallbladder in: (one-day post hatching -1dph); (1-month post hatching - 1mph); and (4mph): **(a,d,g)** chicken; **(b,e,h)** ducks; **(c,f,i)** pigeon. Liver (L), Stomach (S), and Intestine (I). Right lobe (R) having the gall bladder (GB), left lobe (LL) is divided in chicken only, intermediate process (white arrow) and spleen (SP). Scale bar = 1 cm.

**Figure 2:**
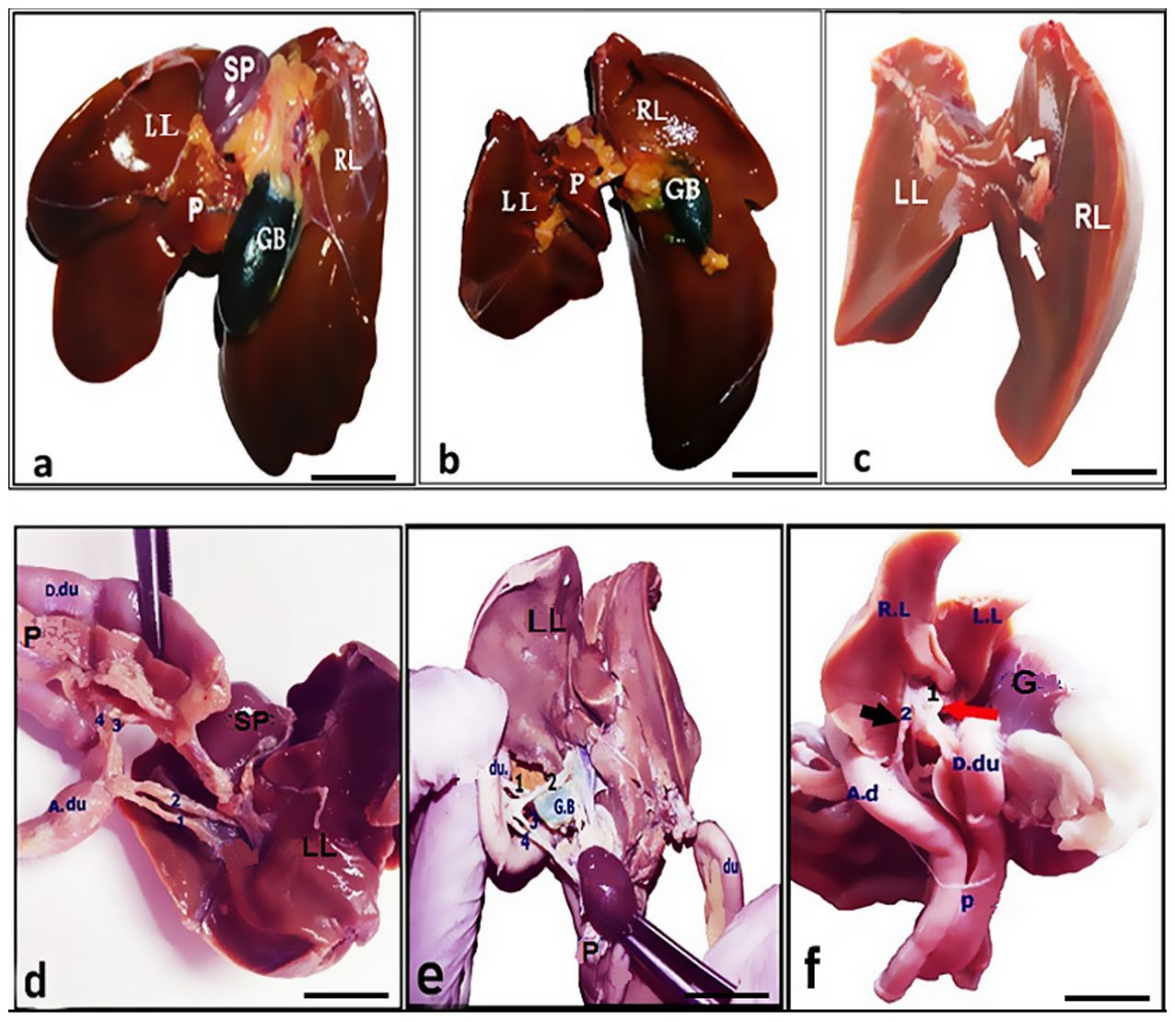
Photograph of the visceral surface of the liver showing the gallbladder in adult age at 8-months post hatching (8mph): **(a)** chicken; **(b)** ducks; **(c)** pigeon. Right lobe (R) having the gall bladder (GB), left lobe (L) is divided in chicken only, intermediate process (P and white arrow) and spleen (SP). Also, visceral surface of liver showing extra hepatic bile ducts: **(d)** in chicken; cystico-enteric duct (1) arise from gall bladder (G.B), hepato-enteric duct (2) arise from the liver and the two pancreatic ducts (3,4), they open in the disital limb of the ascending limb of the duodenum (A.du), descending limb of the duodenum (D.du) and the pancreas (panc.), spleen (SP). **(e)** in ducks, cystico-enteric duct (1), hepato-enteric duct (2) and the two pancreatic ducts (3,4), they open in the ascending limb of the duodenum (A.du.), pancreas (panc.), spleen (SP), gallbladder (G.B). **(f)** in pigeon, the left hepato-enteric duct (1) and right hepato-enteric duct (2), one pancreatic duct (arrow) right lobe (R.L) and left lobe (L.L) of liver, Gizzard **(g)**, ascending duodenum(A.du) and desending duodenum (D.du) and pancreas (p). Scale bar = 1 cm.

**Table 1:** Morphometric parameters of the gallbladder and liver during post-hatching development in chicken and duck (Mean ± SD, *n* = 4).

Parameter	Species	1 dph	1 mph	4 mph	8 mph
Total body weight (g)	Chicken Duck	31.2 ± 2.1ᵃ 42.3 ± 3.0ᵃ	125 ± 15ᵇ 325 ± 28ᵇ	1,200 ± 85ᶜ 1,500 ± 95ᶜ	1,600 ± 110ᵈ 2000 ± 140ᵈ
Gallbladder length (mm)	Chicken Duck	8.1 ± 0.6ᵃ 6.2 ± 0.5ᵃ	15.4 ± 1.0ᵇ 11.7 ± 0.8ᵇ	22.1 ± 1.6ᶜ 15.5 ± 1.2ᶜ	28.8 ± 2.0ᵈ 20.1 ± 1.5ᵈ
Gallbladder width (mm)	Chicken Duck	2.3 ± 0.2ᵃ 3.6 ± 0.3ᵃ	5.7 ± 0.5ᵇ 3.8 ± 0.4ᵇ	9.7 ± 0.9ᶜ 6.8 ± 0.7ᶜ	14.7 ± 1.5ᵈ 10.6 ± 1.1ᵈ
Gallbladder weight (g)	ChickenDuck	0.08 ± 0.01ᵃ 0.06 ± 0.01ᵃ	0.32 ± 0.03ᵇ 0.25 ± 0.02ᵇ	0.91 ± 0.08ᶜ 0.68 ± 0.06ᶜ	1.45 ± 0.13ᵈ 1.11 ± 0.10ᵈ
Empty gallbladder weight (g)	Chicken Duck	0.06 ± 0.01ᵃ 0.05 ± 0.01ᵃ	0.22 ± 0.03ᵇ 0.20 ± 0.02ᵇ	0.61 ± 0.08ᶜ 0.52 ± 0.06ᶜ	1.15 ± 0.13ᵈ 1.09 ± 0.10ᵈ
Bile volume (mL)	Chicken Duck	0.02 ± 0.03ᵃ 0.01 ± 0.05ᵃ	0.15 ± 0.03ᵇ 0.05 ± 0.04ᵇ	0.31 ± 0.08ᶜ 0.18 ± 0.06ᶜ	0.85 ± 0.13ᵈ 0.75 ± 0.10ᵈ
Liver weight (g)	Chicken Duck	1.15 ± 0.87ᵃ 1.86 ± 0.43ᵃ	5.15 ± 0.6ᵇ 16.36 ± 0.53ᵇ	35.69 ± 0.03ᶜ 46.53 ± 0.66ᶜ	38.67 ± 2.1ᵈ 54.45 ± 3.2ᵈ
GB/Liver weight ratio (%)	Chicken Duck	6.96 3.23	6.21 1.53	2.55 1.46	3.75 2.04

*ᵃ*^−^ᵈ Values within the same row with different superscripts differ significantly (*p* < 0.05).

#### 3.1.2 Duck (*Anas platyrhynchos*)

In all examined ducks of all age groups (*n* = 16) the gallbladder was constantly observed. It occupied a deep cystic fossa on the visceral surface of the right liver lobe and did not extend to the ventral hepatic border. It was ovoid with a cranially directed apex and contained dark green bile (Figures 1b,e,h, 2b; Table 1). The ductal arrangement was similar to that in chickens: a hepato-cystic duct, a Cystico-enteric duct, and a hepato-enteric duct. The Cystico-enteric and hepato-enteric ducts opened with two pancreatic ducts into the distal ascending duodenum (Figure 2e).

#### 3.1.3 Pigeon (*Columba livia*)

As the pigeon lacks a gallbladder, histological examination was restricted to the extrahepatic bile ducts, which showed no cystic fossa on the right liver lobe (Figures 1c,f,i, 2c). Consequently, the Cystico-enteric duct was absent. The biliary drainage was accomplished by two hepato-enteric ducts. The right hepato-enteric duct drained the right liver lobe and opened into the middle portion of the ascending duodenum. The left hepato-enteric duct drained the left liver lobe and opened into the proximal descending duodenum. A single pancreatic duct opened into the ascending duodenum distal to the right hepato-enteric duct opening (Figure 2f).

### 3.2 Histological and histochemical changes during post-hatching development

Light microscopic examination revealed that the gallbladder wall in both species (Chickens and Ducks) was composed of four distinct tunics: mucosa, lamina propria-submucosa, muscularis, and serosa/adventitia. Age-dependent histological maturation was evident in both species. (Figures 3–6).

**Figure 3:**
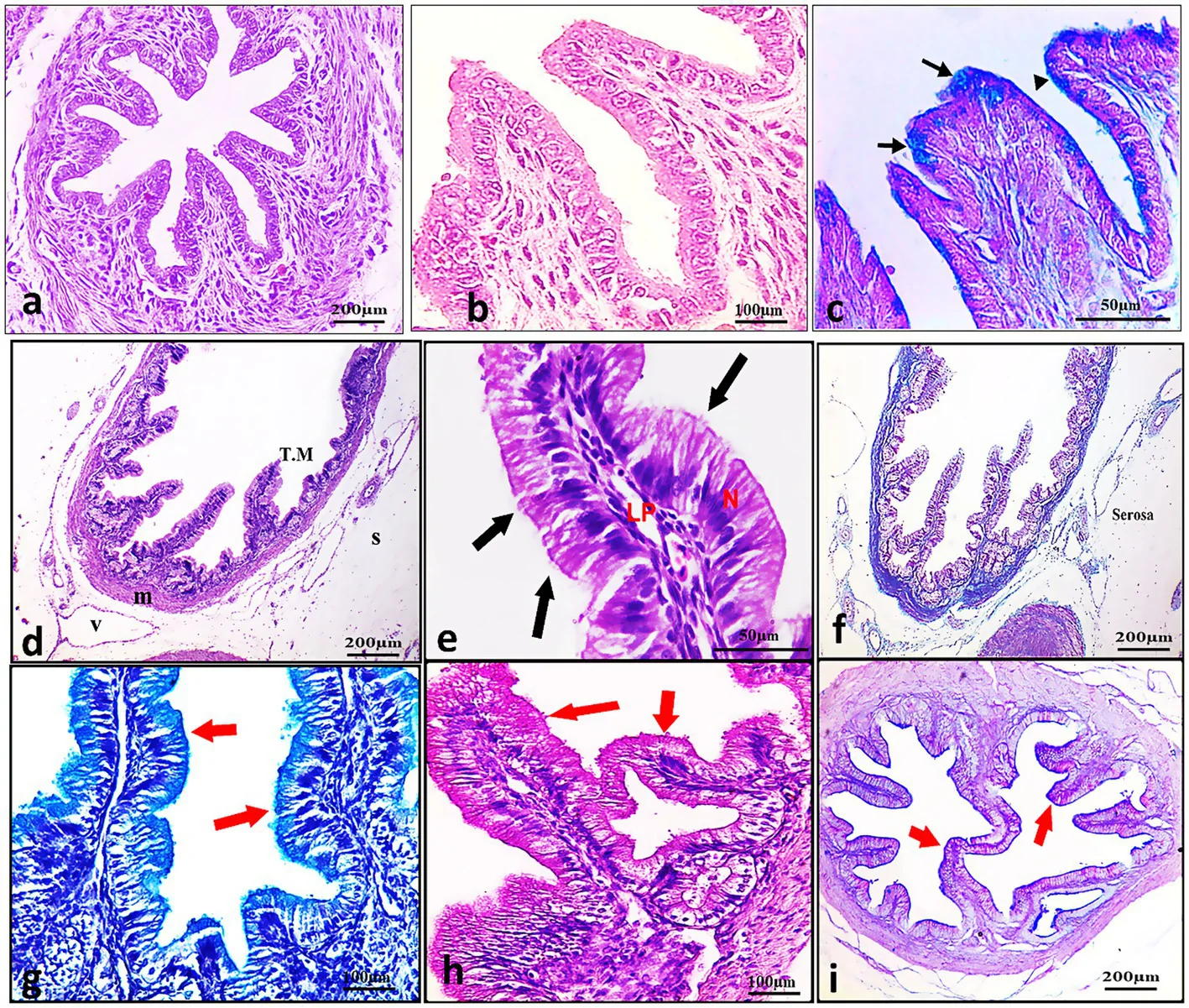
Photomicrographs of transverse sections of the gallbladder at 1-day post-hatching (1 dph) in chicken **(a-c)** and duck (**d-i**). **(a)** H&E stain showing the wall layers of the gallbladder in chicken. **(b)** Higher magnification of **(A)** showing intense PAS-positive reaction in the apical cytoplasm of the surface epithelium. **(c)** Positive Alcian blue (pH 2.5) reaction in the surface epithelium. **(d)** H&E stain showing the wall layers of the gallbladder in duck. **(e)** Higher magnification of (d) showing the simple columnar epithelium (black arrow). **(f)** Masson’s trichrome stain demonstrating collagen fibers in the lamina propria-submucosa and serosal layers. **(g)** Strong positive Alcian blue (pH 2.5) reaction (red arrow of apical surface). **(h)** Strong positive PAS reaction (red arrow of apical surface). **(i)** Combined PAS-AB (pH 2.5) stain showing strong positive reaction. T.M, Tunica mucosa; m, muscle layer; s, serosal layer; v, vein. (blue arrow).

**Figure 4:**
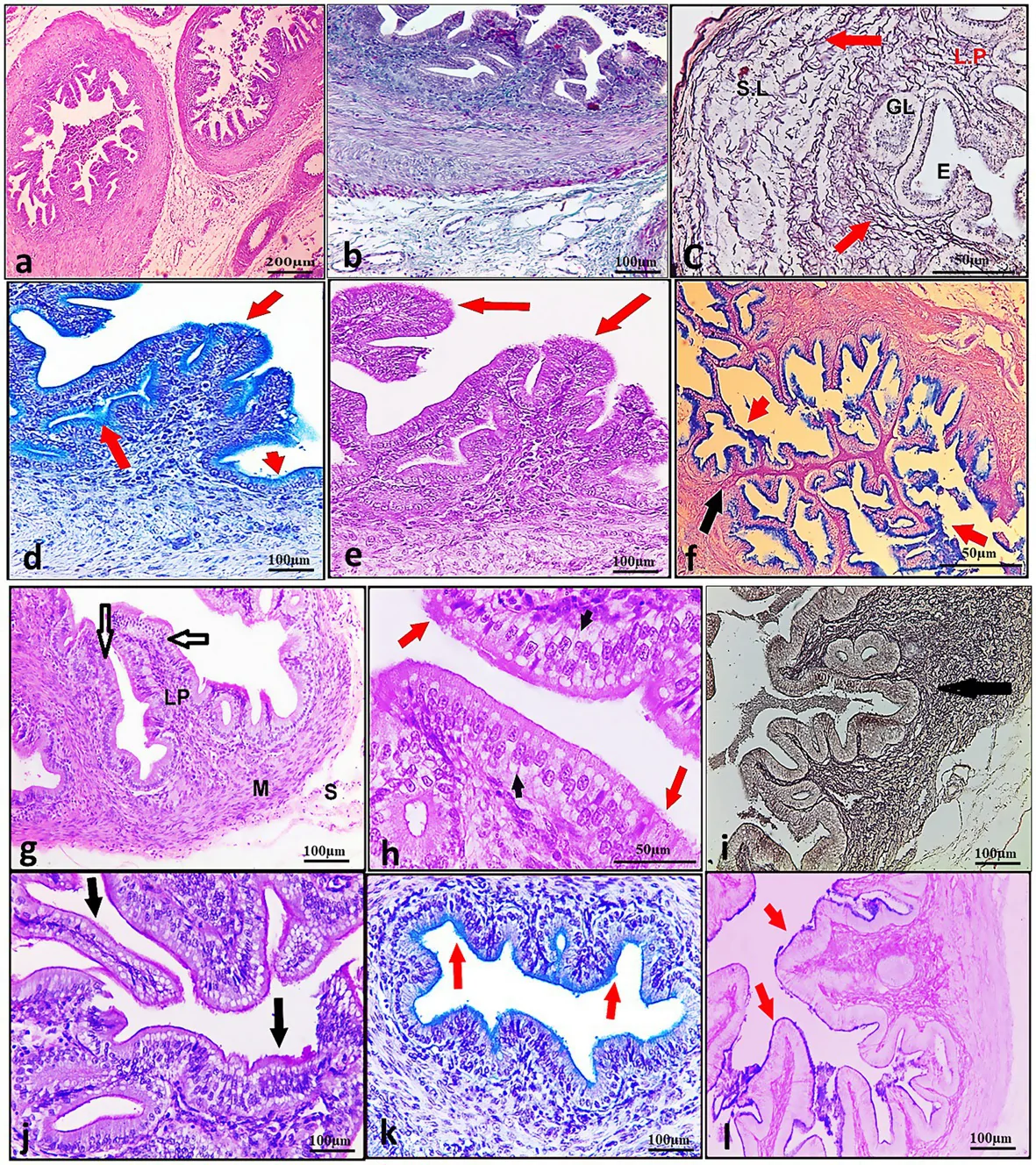
Photomicrographs of transverse sections of the gallbladder at 1-month post-hatching (1 mph) in chicken **(a-f)** and duck **(g-i)**. **(A)** H&E stain showing the wall layers of the gallbladder in chicken. **(b)** Masson’s trichrome stain demonstrating collagen fibers in the lamina propria-submucosa and serosal layers. **(c)** Gomori’s reticulin impregnation demonstrating reticular fibers (red arrows) in the lamina propria-submucosa and serosal layers. (d) Strong positive Alcian blue (pH 2.5) reaction in the apical surface of the epithelium (red arrow). **(e)** Strong positive PAS reaction in the apical surface of the epithelium (red arrow). **(f)** Combined PAS-AB (pH 2.5) stain demonstrating mixed neutral and acidic mucins (red arrows). **(g)** H&E stain showing the wall layers of the gallbladder in duck and the mucosal epithelim (black arrow). **(h)** Higher magnification of **(g)** showing the simple columnar epithelium (red arrows) with vacuolated cytoplasm (black arrow). **(i)** Gomori’s reticulin impregnation demonstrates reticular fibers in the lamina propria-submucosa and serosal layers (black arrow). **(j)** Strong positive PAS reaction in the apical surface of the epithelium (red arrow). **(k)** Strong positive Alcian blue (pH 2.5) reaction in the apical surface of the epithelium (red arrow). **(l)** Combined PAS-AB (pH 2.5) stain showing strong positive reaction. T.M, Tunica mucosa; E, Epithelium; Lp, lamina propria; GL, glands; SL, Submucosal layer; m, muscle layer; s, serosal layer.

**Figure 5:**
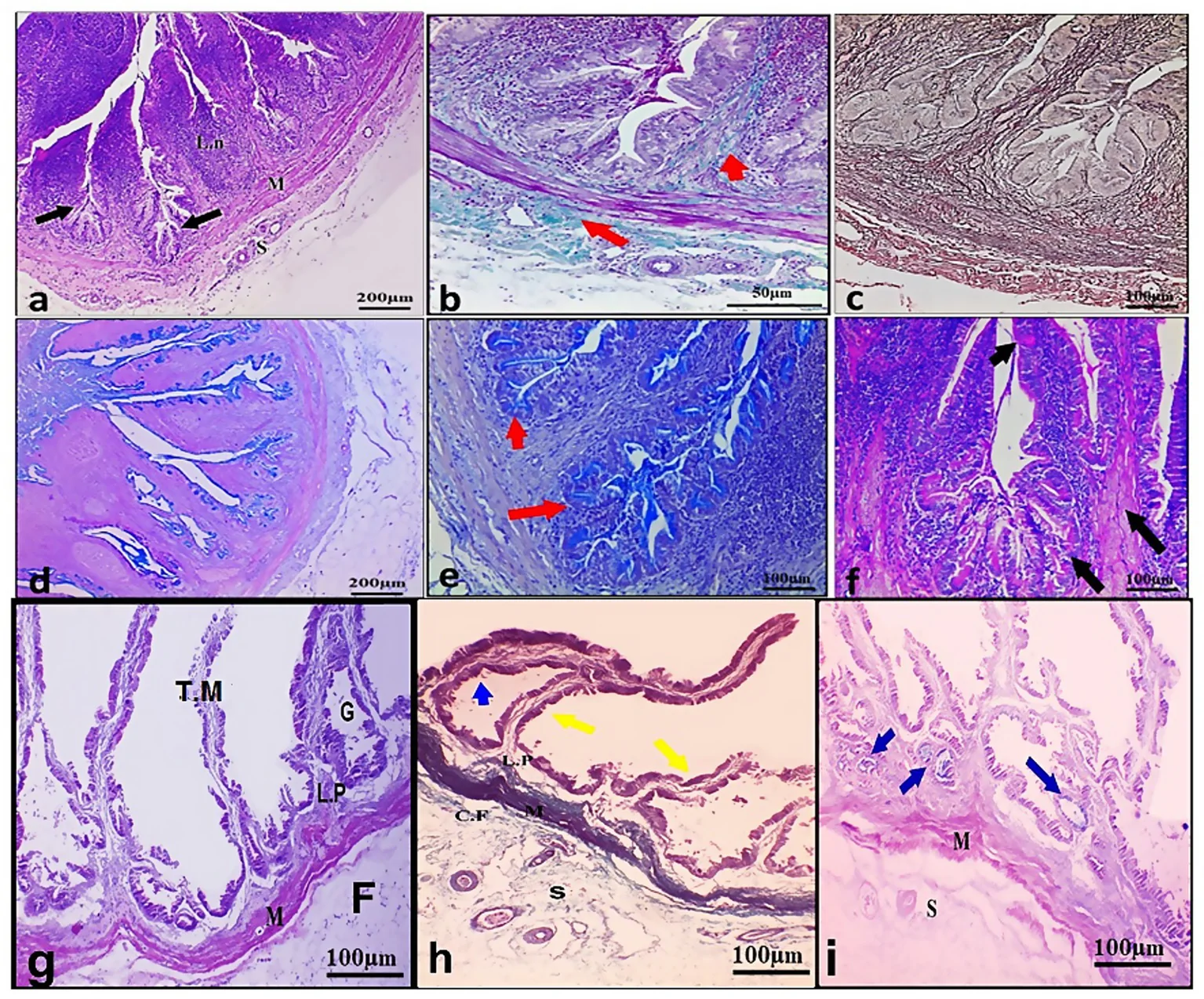
Photomicrographs of transverse sections of the gallbladder at 4 months post-hatching (4 mph) in chicken **(a-f)** and duck **(g-i)**. **(a)** H&E stain showing the wall layers of the gallbladder with tubuloalveolar glands in chicken (black arrow). **(b)** Masson’s trichrome stain demonstrating collagen fibers in the lamina propria-submucosa and serosal layers (red arrow). **(c)** Gomori’s reticulin impregnation demonstrating reticular fibers in the lamina propria-submucosa and serosal layers. **(d)** Strong positive Alcian blue (pH 2.5) reaction in the apical surface of the epithelium. **(e)** Higher magnification of **(d)** showing positive acidic mucin in tubuloalveolar glands (red arrow). **(f)** Strong positive PAS reaction in the apical surface of the epithelium (black arrow). **(g)** H&E stain showing the wall layers of the gallbladder in duck. **(h)** Masson’s trichrome stain demonstrating collagen fibers in the lamina propria-submucosa and serosal layers, mucosal epithelium (yellow arrow) and simple tubular gland-like invaginations (blue arrow). **(i)** Moderate positive combined PAS-AB (pH 2.5) stain (blue arrow). T.M, Tunica mucosa; E, Epithelium; Lp, lamina propria; G, glands; ln, lymphoid nodules; m, muscle layer; s, serosal layer, C.F, collagen fibers; F, Fatty tissue.

**Figure 6:**
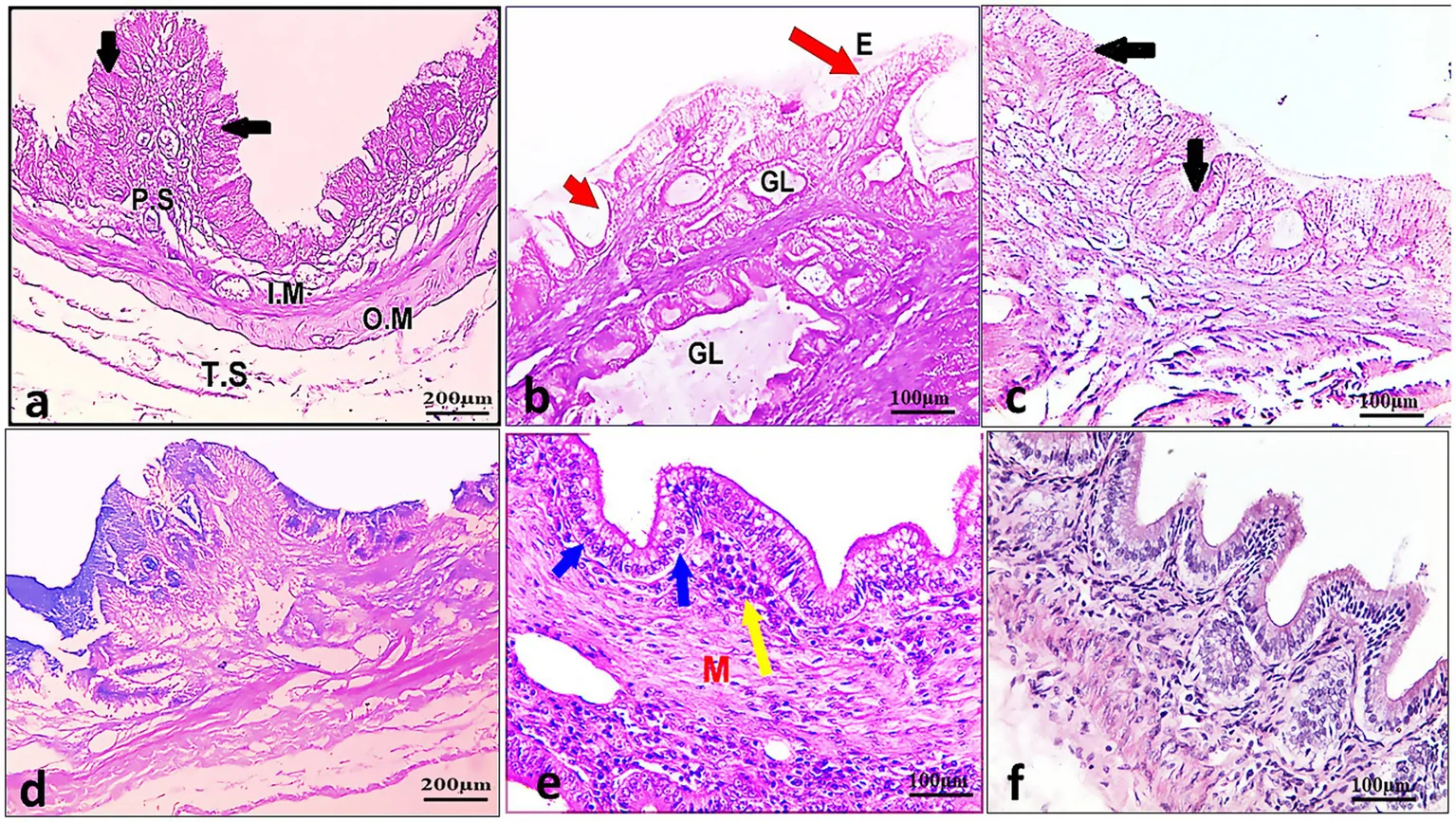
Photomicrographs of transverse sections of the gallbladder at 8 months post-hatching (8 mph) in chicken **(a–d)** and duck **(e,f)**. **(a)** H&E stain showing the wall layers and mucosal epithelium (black arrow) of the gallbladder in chicken. **(b)** H&E stain showing mucosal epithelium (red arrow) and submucosal gland **(c)** positive PAS reaction in the apical surface of the epithelium (black arrow). **(d)** positive Alcian blue (pH 2.5) reaction in the apical surface of the epithelium **(e)** H&E stain showing the wall layers of the gallbladder in duck showing mucosal of simple columnar epithelium (blue arrow) with some diffuse lymphatic tissue (yellow arrow). **(f)** Weigerts elastic tissue stain demonstrating elastic fibers in the lamina propria-submucosa and serosal layers. T.M, Tunica mucosa; E, Epithelium; ps, propria-submucosa; Gl, glands; I.M, inner circular muscular layer; O.M, outer longitudinal muscle layer; T.S, tunica serosal; M, submucosal connective tissue.

#### 3.2.1 Stage I: one day post-hatching (1 dph)

At 1-day post-hatching, the gallbladder mucosa in both chickens (Figures 3a–c) and ducks (Figures 3d–i) formed simple longitudinal villus-like folds. In chickens, the lining epithelium was composed of low columnar cells with ovoid, basally located vesicular nuclei. In contrast, the duck epithelium consisted of tall simple columnar cells with large, oval, basally located vesicular nuclei. The lamina propria-submucosa was a thin layer of loose connective tissue rich in collagen and reticular fibers in both species. The tunica muscularis consisted of a single thin layer of circularly arranged smooth muscle fibers. Histologically, the apical cytoplasm of epithelial cells in both species showed intense PAS and Alcian blue (pH 2.5) positivity, confirmed by combined AB-PAS staining. This indicates constitutive secretion of mixed neutral and acidic mucins from day one post-hatching.

#### 3.2.2 Stage II: one-month post-hatching (1 mph)

Mucosal complexity increased in both species. In chickens (Figures 4a–f) and in ducks (Figures 4g–l) compared to 1 dph. In chickens, the mucosal folds became thicker and branched, lined by simple columnar epithelium. The epithelial cytoplasm was eosinophilic with basally located nuclei. In ducks, the primary folds became more prominent and exhibited secondary branching. The epithelium was tall columnar and displayed extensive apical cytoplasmic vacuolation, suggestive of active secretory or absorptive function. Notably, simple tubular gland-like invaginations of the epithelium into the underlying lamina propria-submucosa were observed in both species at this stage. Masson’s trichrome and Gomori’s reticulin stains revealed a distinct distribution of collagen and reticular fibers in the lamina propria-submucosa, intermuscular connective tissue, and serosal layer. Microscopic evaluation stated that the apical epithelial cytoplasm maintained intense PAS and Alcian blue (pH 2.5) positivity, indicating continued secretion of mixed neutral and acidic mucins.

#### 3.2.3 Stage III: four months post-hatching (4 mph)

At 4 months post-hatching, significant species-specific differentiation was evident. In chickens (Figures 5a–f) and in ducks (Figures 5g–i). In chickens, the mucosa was thinner and exhibited longer, branched villus-like folds. A key finding was the differentiation of the tunica muscularis into two distinct layers: a thick inner circular layer and a thinner outer longitudinal layer of smooth muscle. The lamina propria-submucosa contained numerous lymphoid nodules and compound tubuloalveolar glands, which were absent in ducks. Both the surface epithelium and the submucosal glands in chickens showed intense positive reactions to PAS and Alcian blue (pH 2.5), indicating secretion of mixed neutral and acidic mucins. In ducks, the mucosal folds were also thinner and exhibited longer villus-like folds, similar to chickens. However, the tunica muscularis remained unilaminar, consisting of a single thick circular smooth muscle layer. Lymphoid nodules were sparse and submucosal glands were not observed. Microscopically, the duck epithelium showed moderate PAS and Alcian blue positivity.

#### 3.2.4 Stage IV: eight months post-hatching (8 mph)

At 8 months post-hatching, the gallbladder reached full histological maturity in both species. In chickens (Figures 6a–d) and in ducks (Figures 6e,f). The wall was at its maximum thickness, and the mucosa displayed species-specific folding patterns. In chickens, the mucosa exhibited long, complex, branched villus-like folds, whereas in ducks the mucosal folds were comparatively short. The tunica muscularis was significantly thicker than at all previous stages in both species. The serosa was the thickest layer, composed of loose connective tissue containing arteries, veins, nerves, and adipocytes. Submucosal glands were prominent in chickens but were not observed in ducks. Notably, the lymphoid nodules observed at 4 mph in chickens were absent at 8 mph, with no lymphoid aggregates detected but there were some diffuse lymphatic tissues observed in lamina propria. Histochemically, both the surface epithelium and submucosal glands in chickens maintained intense PAS and Alcian blue (pH 2.5) positivity, while the duck epithelium showed moderate reactivity. Significant species-specific and age-dependent differences were observed in the gallbladder wall of chickens and ducks. These differences are summarized in Table 2.

**Table 2:** Age-related histological and histochemical differences in the gallbladder wall between chichi and duck dph, day post-hatching. Mph, month post-hatching. CT, connective tissue. +ve, positive.

Parameter	Stage I: 1 dph	Stage II: 1 mph	Stage III: 4 mph	Stage IV: 8 mph
Mucosal folds				
Chicken	Simple, longitudinal, villus-like	Thicker, branched	Longer, branched, thinner mucosa	Long, complex, branched
Duck	Simple, longitudinal, villus-like	Primary folds with secondary branching	Longer, villus-like, thinner mucosa	Short
Lining epithelium				
Chicken	Low columnar, ovoid basal nuclei	Simple columnar, eosinophilic cytoplasm	Simple columnar	Simple columnar
Duck	Tall columnar, large oval basal nuclei	Tall columnar, apical vacuolation	Simple columnar	Simple columnar
Tunica muscularis				
Chicken	Single thin circular layer	Single thin circular layer	Bilaminar: thick inner circular + thin outer longitudinal	Thicker bilaminar
Duck	Single thin circular layer	Single thin circular layer	Unilaminar: single thick circular	Thicker unilaminar
Lamina Propria-Submucosa				
Chicken	Thin, loose CT	Tubular gland-like invaginations	Lymphoid nodules + Compound tubuloalveolar glands	Prominent submucosal glands, diffuse lymphatic tissue
Duck	Thin, loose CT	Tubular gland-like invaginations	Sparse lymphoid nodules, no glands	No submucosal glands, diffuse lymphatic tissue
Histochemistry PAS/AB pH2.5				
Chicken	Intense +ve, mixed mucins	Intense +ve	Intense +ve in epithelium and glands	Intense +ve in epithelium and glands
Duck	Intense +ve, mixed mucins	Intense +ve	Moderate +ve	Moderate +ve

## 4 Discussion

This work provides the first comprehensive timeline of post-hatching gallbladder development in domestic chickens and ducks and compares it with the absence of gallbladder in pigeons. Our results are built on previous studies of the morphology of adult birds and help to clarify species-specific adaptations of the biliary system.

### 4.1 Gross morphology and biliary duct system

The continued presence of a gallbladder in chickens and ducks and its absence in pigeons confirms previous reports (1, 2). The tubular morphology in chicken and the ovoid shape in duck filling a deep cystic fossa agrees with the descriptions of (7). This morphological divergence probably parallels functional requirements: the elongated chicken gallbladder may allow storage of bile in a granivorous species with intermittent feeding, while the compact duck gallbladder is adapted to a more continuous feeding style (18). The extrahepatic ductal arrangement seen here in hepato-cystic, Cystico-enteric and hepato-enteric ducts in chickens and ducks is consistent with the classic avian pattern. As mentioned earlier in columbiform, the dual hepato-enteric ducts in pigeons compensate for the lack of gallbladder. Recent comparative work on Aseel chickens confirms that this tri-ductal system is conserved between breeds although duct diameter increases significantly with age, supporting our observation of post-hatching maturation (18, 19). Diet has long been suggested to be the main driver of gallbladder presence. Our data support the hypothesis that species with intermittent feeding retain a gallbladder for the concentration and store of bile, while continuous feeders such as pigeons depend on direct hepatic secretion. However, recent phylogenetic analyses indicate that loss of the gallbladder in Aves has taken place on multiple independent occasions and may be associated with body mass and metabolic rate, as well as diet. This justifies further investigation beyond morphological studies (20–22). The GB/Liver ratio decreased significantly after 1 month in both species, indicating allometric growth of the liver relative to the gallbladder.

### 4.2 Histological post-hatching development

This is one of the first studies describing age-dependent histogenesis of avian gallbladder from 1 dph to 8 mph. The absence of a lamina muscularis mucosae at all stages is in agreement with previous descriptions in adult fowl and confirms the absence of this layer in the avian gallbladder as in mammals (23, 24). One of the main findings was the progressive increase in the mucosal complexity, stromal collagenization and muscularis thickness with age (Table 3). At 1 dph, the simple folds developed into branched primary and secondary folds at 4–8 mph, correlating with the functional maturation of the digestive tract. The significant increase in epithelial height from 1 dph to 8 mph suggests an increase in absorptive and secretory capacity, probably in adaptation to dietary changes from yolk dependence to exogenous feeding (25, 26). A novel observation is that the muscularis was differentiated into inner circular and outer longitudinal layers in chickens by 8 mph, but not in ducks. This suggests motility adaptations in these species. The bilaminar arrangement in chickens may allow for more forceful emptying, in keeping with their elongated morphology. In ducks, however, the ovoid sac may be sufficient with the single thick circular layer. Similar species-specific patterns of muscular activity have been observed in the quail and guinea fowl (27, 28). The lymphatic infiltration seen at 4–8 mph suggests an immunological maturation of the biliary mucosa. The age-dependent lymphoid development is probably contributing to mucosal defense, with the gallbladder as a potential entry site for enteric pathogens, as recently suggested for avian gut-associated lymphoid tissue (29). At 4mph chickens had fully developed tubule-alveolar glands and ducks had primitive gland-like invaginations, suggesting accelerated glandular maturation in chickens. Chickens, at 4 mph, have a bilaminar tunica muscularis, which provides the structural basis for a more efficient gallbladder contraction than the unilaminar tunica muscularis of ducks. High bile volume and intense mucin secretion in chickens are associated with the absence of submucosal glands, suggesting that submucosal glands may be involved in bile modification. The rapid development of GALT in chickens at 4 mph, in contrast to the lack of GALT in ducks, suggests fundamental differences in mucosal immune strategies between the two species.

**Table 3:** Histomorphometrical parameters of the gallbladder wall during post-hatching development in chicken and duck (Mean ± SD, *n* = 4).

Parameter	Species	1 dph	1 mph	4 mph	8 mph
Epithelial height (μm)	Chicken Duck	12.4 ± 1.2ᵃ 14.5 ± 1.8ᵃ	15.7 ± 1.5ᵇ 19.5 ± 2.3ᵇ	24.3 ± 2.1ᶜ 33.2 ± 2.7ᶜ	28.9 ± 2.4ᵈ 35.8 ± 3.1ᵈ
Villus length (μm)	Chicken Duck	95.3 ± 7.2ᵃ 112.7 ± 9.8ᵃ	153.6 ± 12.1ᵇ 186.2 ± 15.3ᵇ	225.8 ± 18.4ᶜ 284.5 ± 22.1ᶜ	315.3 ± 19.7ᶜ 234.4 ± 16.9ᵈ
Lamina propria thickness (μm)	Chicken Duck	18.6 ± 2.1ᵃ 24.2 ± 2.5ᵃ	35.3 ± 4.2ᵇ 38.8 ± 4.8ᵇ	44.7 ± 7.5ᶜ 55.1 ± 8.2ᶜ	62.8 ± 11.3ᵈ 58.6 ± 10.5ᵈ
Muscularis thickness (μm)	Chicken Duck	83.08 ± 6.86ᵃ 85.08 ± 6.86ᵃ	87.01 ± 5.96ᵇ 91.27 ± 6.21ᵇ	94.15 ± 3.76ᶜ 110.13 ± 6.81ᶜ	98.40 ± 6.13ᵈ 119.68 ± 9.78ᵈ

ᵃ^−^ᵈ Means within the same row with different superscripts differ significantly (*p* < 0.05).

At 8 mph, chickens developed significantly longer and more complex villus-like folds (315.3 ± 19.7 μm) than ducks (234.4 ± 16.9 μm), in conjunction with the persistence of submucosal glands and intense mucin secretion. This means the adult chicken has a larger surface area for both absorption and secretion. Collagen, reticular and elastic fibers were present in the connective tissue stroma of the gallbladder wall at all the developmental stages studied. This triad of fiber provides tensile strength, structural support, and resilience, respectively. The age-related increase in fiber density correlates with the observed thickening of the wall and likely enhances the organ’s capacity to accommodate varying bile volumes. The absence of a lamina muscularis mucosae at all stages agrees with previous descriptions (30). The disappearance of lymphoid nodules in chickens at 8 mph suggests that gallbladder-associated lymphoid tissue is transient and may function primarily during juvenile stages. The persistence of submucosal glands exclusively in chickens represents a definitive species-specific histological marker at maturity.

### 4.3 Histochemical properties of mucin

A central finding is the high intensity of constitutive PAS and Alcian blue positivity at all post-hatching stages. In contrast to the mammalian gallbladder where mucin secretion is often stimulus dependent, the avian epithelium appears to maintain a continuous mucin barrier from 1 dph. This persistent layer of neutral and acidic mucopolysaccharides likely protects the epithelium from bile salt cytotoxicity and bacterial translocation, which is of particular importance in precocial birds exposed to environmental pathogens immediately post hatch (30). The presence of tall columnar epithelium with mixed mucin secretion in ducks at 1 dph indicates an early adaptation for higher absorptive and protective functions as compared to chicken. The apical localization of mucins in the absence of true goblet cells is consistent with the hypothesis that avian columnar epithelial cells are multifunctional absorptive and secretory cells. Recent studies have confirmed Muc5ac gene expression in chicken gallbladder epithelium supporting our histochemical data. The minor reduction in AB staining intensity observed in 4 mph ducks requires further investigation but may represent transient changes in mucin sulfation during rapid growth (31–33). The shift to moderate mucin reactivity in ducks at 4 mph, concomitant with increased bile volume, is suggestive of a shift from the process of mucus-dependent concentration to volume-dependent storage. The strong mucin reactivity from 1 dph in both species confirms the secretory nature of the gallbladder from hatching. Despite lower bile volume in ducks, comparable AB-PAS intensity between species suggests that mucins regulate bile concentration, not volume (34). Vacuolated cytoplasm in duck epithelium at 1 mph might be related to increased absorptive activity together with low bile volume.

### 4.4 Limitations and future directions

This study was limited to light microscopy. Ultrastructural and immunohistochemical analyses of mucin types are needed to fully characterize the protective barrier. Additionally, functional studies correlating bile composition with histological maturity would clarify the physiological significance of the changes described (33).

## 5 Conclusion

Chickens and ducks undergo significant post-hatching maturation from 1 dph to 8 mph, characterized by increased villous complexity, stromal thickening, and lymphocytic infiltration. The persistent PAS and Alcian blue positivity confirm a protective mucin barrier from the earliest stage. The absence of a gallbladder in pigeons was associated with a modified bile duct arrangement. These species and age-dependent findings provide a necessary histological baseline for interpreting biliary pathology in avian models.

## Data Availability

The raw data supporting the conclusions of this article will be made available by the authors, without undue reservation.

## References

[1] MacLean RA Beaufrère H Miller E Fowler M. Gruiformes (Cranes, Limpkins, Rails, Gallinules, Coots, Bustards). St. Louis, MO: Elsevier Health Sciences (2014).

[2] Naude AD. Morphology of the Southern Ground-Hornbill (*Bucorvus leadbeateri*) Gastro-Intestinal Tract [master's thesis]. Pretoria: University of Pretoria (2020).

[3] Oldham-Ott CK Gilloteaux J. Comparative morphology of the gallbladder and biliary tract in vertebrates: variation in structure, homology in function and gallstones. Microsc Res Tech. (1997) 38:571–97. doi: 10.1002/(SICI)1097-0029(19970915)38:6<571::AID-JEMT3>3.0.CO;2-I, 9330347

[4] Aboghanima ZK Elsabbgh Y Abdelmoneam A Aboelnour A Nomir AG Elsharaby AA. Morphological and topographical characteristics of the liver of Pekin ducks (*Anas platyrhynchos domesticus*) using computed tomography and cross-sectional anatomy. Damanhour J Vet Sci. (2025) 13:66–81. doi: 10.21608/djvs.2025.383768.1154

[5] Bailey TA Mensah-Brown EP Samour JH Naldo J Lawrence P Garner A. Comparative morphology of the alimentary tract and its glandular derivatives of captive bustards. J Anat. (1997) 191:387–98. doi: 10.1046/j.1469-7580.1997.19130387.x, 9418995 PMC1467696

[6] Purushottam Meshram B Kumari A Kamble M. Histomorphological studies on the gallbladder in guinea fowl (*Numida meleagris*). J Vet Anat. (2021) 33:130–131. Available online at: https://epubs.icar.org.in/index.php/IJVA/article/view/120506

[7] Higashiyama H Kanai Y. Comparative anatomy of the hepatobiliary systems in quail and pigeon, with a perspective for the gallbladder-loss. J Vet Med Sci. (2021) 83:855–62. doi: 10.1292/jvms.20-0669, 33814520 PMC8182306

[8] McLelland J. A Colour Atlas of Avian Anatomy. London: Wolfe Publishing Ltd (1990).

[9] Yilmaz B Demircioglu I Korkmaz D Alan A Yilmaz R Ciris A. Macroanatomic, light and scanning electron microscopic structure of the pecten oculi in northern bald ibis (*Geronticus eremita*). Anat Histol Embryol. (2021) 50:373–8. doi: 10.1111/ahe.12641, 33274793

[10] Mobini B Faradonbeh KS. Histology of extrahepatic bile ducts in the Iranian chukar partridge (*Alectoris chukar*). Thai J Vet Med. (2012) 42:537–41. doi: 10.56808/2985-1130.2436

[11] Mobini B. Histological study of the gall bladder of the common quail (*Coturnix coturnix*). Vet Med (Praha). (2014) 59:261–4. doi: 10.17221/7520-VETMED

[12] Lucchese AM Machry MC Kalil AN Oliveira AF Coimbra FJF. "Exploring anatomy, macroscopic and microscopic structures, and physiological functions of the gallbladder". In: Gallbladder Cancer: A Comprehensive Guide to Management. Cham: Springer (2025). p. 1–13.

[13] Hamodi HM Abed AA Taha AM. Comparative anatomical, histological and histochemical study of the liver in three species of birds. Raf J Sci. (2013) 24:12–23. doi: 10.33899/rjs.2013.78754

[14] Pakurar AS Bigbee JW. Digital Histology: An Interactive CD Atlas with Review Text. Hoboken, NJ: John Wiley & Sons (2011).

[15] Maher M. Descriptive anatomy of hepatic and portal veins with special reference to biliary duct system in broiler chickens (*Gallus gallus domesticus*): a recent illustration. Braz J Poult Sci. (2019) 21:eRBCA-2019-0980. doi: 10.1590/1806-9061-2019-0980

[16] Nir I Nitsan Z Mahagna M. Comparative growth and development of the digestive organs and of some enzymes in broiler and egg type chicks after hatching. Br Poult Sci. (1993) 34:523–32. doi: 10.1080/00071669308417607, 8358639

[17] Bancroft JD Gamble M. Theory and Practice of Histological Techniques. 6th ed. Philadelphia, PA: Elsevier Health Sciences (2008).

[18] Alabdallah ZA. Morphological and histological comparison of the internal organs of birds. Clin Rev Case Rep. (2024) 3:1–11. doi: 10.31579/2835-7957/051

[19] Hodges RD. The Histology of the Fowl. London: Academic Press (1974).

[20] Al-Bakri NA Al-Hamawandy DH. Morphological description and histological structure of gall bladder in local adult chicken *Gallus gallus domesticus*. AAJPS. (2020) 1:6–11.

[21] Housset C Chrétien Y Debray D Chignard N. Functions of the gallbladder. Compr Physiol. (2016) 6:1549–77. doi: 10.1002/j.2040-4603.2016.tb00717.x, 27347902

[22] Daniel EE. Neuropeptide Function in the Gastrointestinal Tract. Boca Raton, FL: CRC Press (1991).

[23] Gofur M. Anatomy and histomorphometry of accessory reproductive glands of the black Bengal buck. Eur J Anat. (2015) 19:171–8. doi: 10.14202/vetworld.2016.346-350

[24] Kausar R Qureshi AS Ali MZ Ateeq MK Usman M. Age induced changes in the microscopic anatomy of the digestive system of Japanese quails (*Coturnix japonica*). Biosci Res. (2016) 13:26–31. Available online at www.isisn.org

[25] Alam MB Hiramatsu K. Histological changes of the mucosal epithelium in the chicken intestine during pre- and post-hatching stages. J Poult Sci. (2025) 62:n/a. doi: 10.2141/jpsa.2025004, 39830138 PMC11733151

[26] Dunislawska A Pietrzak E Wishna Kadawarage R Beldowska A Siwek M. Pre-hatching and post-hatching environmental factors related to epigenetic mechanisms in poultry. J Anim Sci. (2022) 100:370. doi: 10.1093/jas/skab370, 34932113 PMC8826992

[27] Abdulqader SA Huwait ARH. Comparative anatomical and histological study of gall bladder in mallard (*Anas platyrhynchos*) and broiler fowl (*Gallus gallus*). Univ Thi-Qar J Agric Res. (2023) 12:15–25. doi: 10.54174/utjagr.v12i2.254

[28] Talab ME Hamedi S Paryani M. Age-dependent changes of gut-associated lymphoid tissue in one to four-month-old turkeys: a histological study. Bulg J Vet Med. (2022) 25:187. doi: 10.15547/bjvm.2020-0082

[29] Ullah K Siddique Q Tasleem F. Probiotics and gut-associated lymphoid tissue (GALT) in fish: mechanism, application and future perspectives. Probiotics Antimicrob Proteins. (2026) 29:1–23. doi: 10.1007/s12602-026-11109-4 42319665

[30] Chia S Adagi J Ate M Uma K. Comparative studies on blood serum profiles of liver enzymes, proteins, total bilirubin and liver organ morphometry of Muscovy ducks. Black Sea J Agric. (2022) 5:167–71. doi: 10.47115/bsagriculture.1032032

[31] Bao Q Wang L Hu X Yuan C Zhang Y Chang G . Developmental changes of duckling liver and isolation of primary hepatocytes. Animals. (2023) 13:1820. doi: 10.3390/ani13111820, 37889689 PMC10252113

[32] Abdellatif AM Farag A Metwally E. Anatomical, histochemical, and immunohistochemical observations on the gastrointestinal tract of *Gallinula chloropus* (Aves: Rallidae). BMC Zool. (2022) 7:61. doi: 10.1186/s40850-022-00161-6, 37170387 PMC10127349

[33] Denbow DM. "Gastrointestinal anatomy and physiology". In: Sturkie's Avian Physiology, 6th Edn. San Diego, CA: Elsevier (2015). p. 337–66.

[34] Zeinab KA Ashraf El Sharaby, AboelnourAsmaa, Mohamed MA. Abumandour Ahmed G.N. A new anatomical insight into the architecture of biliary duct system, gall bladder, and duodenal papilla with the extrahepatic bile ducts of white Pekin duck (*Anas platyrhynchos*). Tissue Cell (2025) 96:102993. doi: 10.1016/j.tice.2025.102993 40472551

